# Evidence-based practice among physiotherapists in India: a nationwide survey of knowledge, attitude, and implementation behavior

**DOI:** 10.7717/peerj.20632

**Published:** 2026-02-04

**Authors:** Mohammad Sidiq, Jyoti Sharma, Balamurugan Janakiraman, Faizan Kashoo, Aksh Chahal, Ruchi Varshney, Sumbul Ansari, Akriti Pandey, Richa Hirendra Rai, Abdulqader Khormi, Imran Khan, Mohammed M. Alshehri, Monira I. Aldhahi

**Affiliations:** 1 Physiotherapy Department, Tishk International University, Erbil, Iraq; 2 Department of Physiotherapy, Galgotias Multi-disciplinary Research and Development Cell (G-MRDC), Galgotias University, Greater Noida, Uttar Pradesh, India; 3 SRM College of Physiotherapy, Faculty of Medicine and Health Sciences, SRM Institute of Science and Technology (SRMIST), Kattankulathur, Tamil Nadu, India; 4 Department of Physical Therapy and Health Rehabilitation, College of Applied Medical Sciences, Majmaah University, Majmaah, AR Riyadh, Saudi Arabia; 5 Ruchi Advanced Physiotherapy and Wellness Clinic, New Delhi, Delhi, India; 6 School of Physiotherapy, Delhi Pharmaceutical Sciences and Research University, New Delhi, Delhi, India; 7 Department of Physical Therapy, College of Nursing and Health Sciences, Jazan University, Jazan, South, Saudi Arabia; 8 Department of Physiotherapy, University of Engineering and Management, Jaipur, Rajasthan, India; 9 Department of Rehabilitation Sciences, College of Health and Rehabilitation Sciences, Princess Nourah bint Abdulrahman University, Riyadh, Saudi Arabia

**Keywords:** Evidence-based practice, Physiotherapy, Knowledge, Attitude, Practice, Implementation behavior

## Abstract

**Background:**

Physiotherapy services are often integrated within the broader healthcare system in India. Unlike in developed countries, physiotherapy is still limited to indirect access and needs a referral from other healthcare providers, which potentially limits physiotherapists. The purpose of this study was to explore the knowledge or skill, attitude, and behavior towards the implementation of evidence-based practice (EBP) in physiotherapy care. In addition, the physiotherapist’s perception of barriers in the implementation of EBP was inquired.

**Methods:**

A cross-sectional study was conducted through an online survey involving two thousand nine hundred and ninety-six physiotherapists from 22 states/union territories in India. A 24-item EBP questionnaire (EBPQ) investigating knowledge, attitude, and behavior domains with a 1 to 7 Likert scale response for each item, with a higher score indicating a favorable response. A linear regression model was used to analyze the relationship between factors and evidence-based practice behavior among physiotherapists.

**Results:**

The mean age of the participants was 35.8 ± 6.2 years, with a work experience of 13.25 ± 6.38 years. The overall EBPQ mean score of Indian physiotherapists was 3.6 ± 0.38, and the mean scores of the domains—knowledge, attitude, and implementation of EBP—were 3.59 ± 0.53, 4.29 ± 0.79, and 3.20 ± 0.62, respectively. The EBP domains were mainly determined by the educational attainment and workplace, which explained 46.1% of the variance. Lack of time and skills was identified as the top barrier influencing EBP among physiotherapists in India. The behavior dimension score of EBPQ is determined by 8 knowledge items and 2 attitude items, which explain 61.2% of the variance.

**Conclusions:**

The Indian physiotherapists reported a positive attitude toward evidence-based practice; however, their knowledge and behavior were observed to be insufficient. Lack of time, limited skills, a shortage of resources, and limitations in applying EBP were reported as the main barriers.

## Introduction

Globally, the World Health Organization upholds health as a human right, and fundamental to it is the sub-specialty of universal health coverage (UHC) (World Health Organization, 2021). UHC emphasizes the expectation of accessing effective quality healthcare services at the right time and place while bearing manageable costs (Ghandour & Truche, 2022; Bertram et al., 2024). Evidence-based practice (EBP) integrates the best available research and patient-centred knowledge, blended with experience in the clinical field (Mishra, 2024; Mittal et al., 2024) and EBP is expected of healthcare professional. Healthcare professionals are expected to practice evidence-based. As a result, by the time the students in the health care profession complete their studies, they should be equally confident in applying evidence-based practice (Olsen et al., 2013). It is a foundation for improving the effectiveness of physiotherapy interventions and enhancing the quality of physiotherapy services (Scurlock-Evans, Upton & Upton, 2014).

World Physiotherapy, previously known as the World Confederation for Physical Therapy (WCPT), advocates physiotherapists to embrace EBP principles with an emphasis on managing patients and communities based on the best available evidence  (Schubert, 2019; Rushton et al., 2011; World Physiotherapy, 2019). While there is an increase in the international trends toward implementing EBP, the awareness and practice of EBP among physiotherapists in India have been less researched, specifically their understanding, recommendations, and compliance (Panhale, Bellare & Jiandani, 2017; Nolan & Bradley, 2008; Majdzadeh et al., 2012). The majority of published literature is concentrated on the integration of EBP in developed Western countries, assessing the barriers and enablers for EBP integration, but has neglected the challenges that are specific to the Indian context, including limited resources, institutional and cultural variations (Scurlock-Evans, Upton & Upton, 2014; ShahAli et al., 2023; Panhale & Bellare, 2015). Although physiotherapy plays a central role in managing non-communicable diseases like pre- and post-operative rehabilitation of sports injuries, post-stroke rehabilitation, cardio-respiratory rehabilitation, movement disorders, women’s health, and musculoskeletal disorders, *etc*., there is a growing belief that EBP use is inconsistent and hence, may lead to inferior patients’ outcomes (Achttien et al., 2013; Mendonça et al., 2022; Lee, Choi & Jeoung, 2022; Zaidi & Ahmed, 2022; Nielsen et al., 2015; Desmeules et al., 2012).

Based on the few published studies in India, the physiotherapists generally hold a favorable attitude and agree that EBP is important (Panhale, Bellare & Jiandani, 2017; Panhale & Bellare, 2015; Thakkar, 2018). At the same time, limited knowledge, skill deficiencies, and non-reliance on the current scientific evidence are observed among the physiotherapists. The barriers to EBP implementation across the literature are limited access to resources, lack of formal training, and lack of support. It is important to understand the knowledge, attitude, and barriers to implementing EBP among physiotherapists in India to set up strategies, improve capacity building, and formulate effective policy with institutional changes required to support sound clinical decision-making and legitimize the profession (Morales-Osorio et al., 2024). This research seeks to address this gap by examining how knowledge of EBP, awareness of its principles, and its implementation by physiotherapists in India respond to the global call for EBP implementation to embrace what is described as the best practice of informed decision-making concerning the realities of the resources that are both relevant as well as desirable. We hypothesize that there is a positive relationship between the knowledge and attitude towards evidence-based practice and implementation behaviour among physiotherapists in India.

### Methods

### Study design, and registrations

A cross-sectional survey was conducted among Indian physiotherapists from various Indian states and union territories who are engaged in the public sector, private hospitals, or self-employed. The ethical approval was obtained from the Departmental Research Committee, Galgotias University (DRC/FEA/94/24), affirming the ethical standards and oversight for the research and the study was prospectively registered with the Clinical Trial Registry India CTRI/2024/09/073590. The study adhered to the principles of Helsinki (World Medical Association, 2009). All the participants provided online consent following the General Data Protection Regulation (GDPR) guideline recommended by the European Union (Plötz & Bekhof, 2018). Data was collected using a structured online Google form. This study was reported according to the Checklist for Reporting Results of Internet E-Surveys (CHERRIES) (Eysenbach & Wyatt, 2002), the Strengthening the Reporting of Observational Studies in Epidemiology (STROBE) and the Consensus-Based Checklist for Reporting of Survey Studies (CROSS) guidelines (Von Elm et al., 2007; Sharma et al., 2021) (Additional file S1) (Von Elm et al., 2007; Bennett et al., 2011; Eysenbach, 2004).

### Study setting

Physiotherapy education in India primarily revolves around bachelor of physiotherapy (BPT), four years academic study with six months of clinical internship, and the Indian Association of Physiotherapists (IAP) is an exclusive professional body. The National Commission for Allied and Healthcare Professions (NCAHP, 2021), and new competency-based curriculum for physiotherapy emphasizes EBP in the profession and practice with scientific rigor in India. The inclusion criteria for participation in this EBP survey were: (1) registered physiotherapists at IAP, (2) Willingness to participate in the survey, and (3) Minimum of 2 years of work experience. Exclusion criteria were: (1) Individuals who did not provide consent to participate, (2) Physiotherapists who is currently not practicing or teaching physiotherapy.

### Target population

According to the IAP (2021), there are an estimated 15,000 registered physiotherapists in India, and the number of practicing physiotherapists is less than 1 per 10,000 population. The majority of them are members of the Indian Association of Physiotherapists (IAP). There was no direct involvement of patients or the public.

#### Data collection procedure, and data storage

We used the EBP survey questionnaire and posted it on the SurveyMonkey website platform (SurveyMonkey, Palo Alto, CA, USA, www.surveymonkey.com) to collect data, and a closed survey targeting the members of the IAP was conducted. The email and contact details of the physiotherapists were secured from the Treasurer’s office, IAP. Prior to posting the survey questions online, the desk office clerk of Galgotias University sent an invitation from their official mail ID to all the IAP members with an explanation of the objectives, justification of the study, and content of the survey. The invitation email also explained about the time needed to complete the EBPQ questionnaire (10 min) and the expected construct of the questionnaire. The EBPQ survey questions were posted from the clerical office on 9 Sept 2024, and two weeks later, the office clerk sent out a reminder email to the IAP members who did not respond. All the responses were collected by the clerical office of the study center, and the responses were treated anonymously. The IAP email list contained 12,445 active mail IDs, and all of them were considered for the study. The survey period ran over a 15 weeks period from Sept 2024 to December 2024. Furthermore, the physiotherapists contacted were encouraged to further distribute the online survey form and invitation to participate. The survey responses were collected and securely stored in an encrypted computer, and the access was restricted to the project PI (MS). The PI concealed and de-identified all the data, including names and email addresses.

### Variables

The online questionnaire form consists of consent-related questions, demographic characteristics (age, sex, educational attainment, region, workplace, designation, and work assigned), a 24-item EBPQ, perceived barriers to EBP items, job description/preference, educational qualifications, and demographic questions. The EBPQ tool was developed and validated by Upton & Upton (2006) to evaluate nurses’ knowledge, attitude, and application of EBP. Since then the EBPQ has been used to measure the EBP among other healthcare professionals including physiotherapists, to inform educational policies or patient care policies in several setups. Each of the 24 items on the questionnaire is scored on a Likert rating scale of 1 to 7, with the highest score indicating higher EBP cognition (*i.e.,* 1 = poor to 7 = best). There are three subscales (behavior/practice (six items), attitude (four items), and knowledge/skills (14 items)), and the average score of each subscale and the total EBPQ score is then calculated for analysis. The questionnaire was seven pages, and each page of the survey contained 6–8 questions, the respondents were requested to enter the responses manually by clicking one exclusive box for each question and they were allowed to review and change their responses if needed using a back button in the survey page. The EBPQ is a widely used, robust questionnaire, with a Cronbach’s alpha coefficient of 0.87, and subdomain Cronbach’s alpha coefficients are 0.85, 0.79, and 0.91 (Upton & Upton, 2006). To ensure the completeness of the responses, mandatory highlights were used for the questions.

### Sample size and *post-hoc* power analysis

We decided to include all the fully completed responses from the members of IAP, considering the feasibility of the online web-based survey cost, and to have a large enough sample, taking into account the number of predictors (participants per predictor) for the regression model, variability of predictors (educational status, job description, workplace *versus* EBP), and subject-to-variable ratio (Brooks & Barcikowski, 2012). A *post-hoc* power analysis was conducted using G*power software for Windows (version 3.1.9.6) to estimate the power achieved for multiple linear regression in this study. The following inputs were used: sample size *n* = 2,996, fixed models, R^2^ deviation from ‘0’, a detectable effect size of f^2^ 0.1 (small effect), level of significance of the odds at 0.05 (α), number of predictors, k (*n* = 6). The estimated achieved *post-hoc* power (1 −β) was 1.00. Therefore, the findings of this study are based on adequately powered samples for the variables and statistical tests chosen.

### Ethical consideration

The first section of the online form included information regarding the time needed to complete this survey (15 min) and mandatory questions related to the consent form on the cover page that explained the purpose of this study, voluntary participation, understanding of risks, the anonymity of personal information, the absence of incentives, the participant’s right to withdraw at any time, and their acceptance to participate in this survey. The study depended on complete response data for its analysis and to stay unbiased. Therefore, we did not consider it necessary to give participants the option to opt-out by item. The participants were also advised to print and/or take a screenshot to retain a copy of the consent page. All the participants in this study filled out the online consent form. The study reports as per the recommended guidelines by the Declaration of Helsinki (World Medical Association, 2009).

### Data analysis

The data was exported from SurveyMonkey and analyzed using Statistical Package for Social Sciences (SPSS) version 23.0 for Windows (IBM Corp., Armonk, NY, USA). The data was descriptively summarized using mean, standard deviation, and frequency with proportions to describe the socio-demographic characteristics, work-related characteristics, distribution over national territory, barriers, and scores of EBPQ. Pearson’s correlation coefficient was calculated between the sub-domains of EBP scores and age to look for a linear relationship. We employed a backward elimination multiple linear regression analysis to identify the influential variables associated with the sub-domains of EBPQ and overall EBPQ average scores. An additional linear regression analysis was performed to identify indicators (items) within the attitude and knowledge domains that significantly contribute to the explaining of behavior/practice domain scores of the EBPQ among the participants. The level of significance for the admission of variables was set at ≤ 0.05 and for the exit at >0.10 to improve model efficiency and reduce potential overfitting. The standardized coefficient (β) with 95% CI was reported with *p*-values and coefficients of determination (R^2^). We also reported results according to the Sex and Gender Equity in Research (SAGER) guidelines (Heidari et al., 2016) (Table S1).

## Results

### Participants

The targeted response was at least 1,000 participants to allow regression analyses with socio-demographic variables and subgroups with sufficient power. Since the exact number of physiotherapists in India is not known, we could not estimate the response rate. Of the emails sent, 2,966 participants completed the survey and were included in the analyses, while 986 respondents did not provide their consent. No information regarding the view rate was gathered.

### Socio-demographic data

The mean age of the participants was 35.8 ± 6.2 years, the mean experience was 13.25 years (SD 6.38), and most of the respondents were female (n 1,872, 62.5%). About 50% of the respondents held master’s degree and a majority of them reported to be working at private physiotherapy colleges (n 733, 24.5%), and community care (n 669, 22.3), followed by hospitals (n 538, 18%). Most participants represented the northern region, southern region, and central region (n 1113 (37.1%), n 768 (25.6%), and n 713 (23.8) respectively). The least representation was from the Eastern and Western regions (n 09 (0.3%) and n 393 (13.2%), respectively). About 70% reported being involved in patient care (Table 1). No significant difference was observed in socio-demographic variables and EBPQ scores between male and female participants (Table S1).

**Table 1:** Demographic charateristics.

**Characteristics**	**Descriptive statistics**
Age in years, mean ± SD	35.83 ± 6.22
Age group, n (%)	
<30 years	648 (21.6)
30–40 years	1792 (59.8)
>40 years	556 (18.6)
Sex, n (%)	
Male	1124 (37.5)
Female	1872 (62.5)
Geographical zones, India, n (%)	
Central zone	713 (23.8)
Eastern zone	09 (0.3)
Northern zone	1113 (37.1)
Southern zone	768 (25.6)
Western zone	393 (13.2)
Educational status	
Diploma	7 (0.2)
Bachelor’s degree	1225 (40.9)
Master’s degree	1470 (49.1)
DPT	22 (0.7)
PhD scholar	11 (0.4)
PhD degree	261 (8.7)
Workplace, n (%)	
Community care	669 (22.3)
Private clinic	341 (11.4)
Nursing home/poly clinic	335 (11.2)
Hospital	538 (18)
Teaching Hospital/university	380 (12.7)
Private college	733 (24.5)
Work experience, mean ± SD	13.25 ± 6.38
Work preference/assigned, n (%)	
Patient care	2076 (69.3)
Teaching	11 60 (38.7)
Administration/managerial	400 (13.4)
Research	287 (9.6)
Knowledge subscale, mean ± SD (1–7)	3.59 ± 0.53
Attitude subscale, mean ± SD (1–7)	4.29 ± 0.79
Practice subscale, mean ± SD (1–7)	3.20 ± 0.62
EBPQ overall, mean ± SD (1–7)	3.61 ± 0.38

**Notes.**

DPT Doctorate of Physiotherapy SD Standard deviation EBPQ Evidence-based practice questionnaire

### EBPQ and subdomain scores

The overall EBPQ mean score of the participants was 3.61 (SD 0.38). Among the subscales, the behavior/practice score was 3.61 (SD 0.38) out of seven, which indicates moderate behavior of EBP. The attitude dimension average score of 4.29 (SD 0.79) indicates a relatively positive attitude toward EBP among the physiotherapists. The mean score of the knowledge dimension was 3.59 (SD 0.53) suggesting a low level of EBP knowledge among the participants. The item-wise analysis of the EBPQ (Table S2 showed that IT skills (item # 12) of knowledge/skills had the low score (mean 3.28, SD 1.3), and monitoring of practice skills (item # 13) demonstrated the highest score (mean 4.77, SD 1.9). Attitude items #7 (“new evidence is important”) and item # 8 (“I welcome questions on my practice”) were observed with minimum (mean 3.85, SD 1.4) and maximum scores (mean 4.59, SD 1.7), respectively. Among the behavior items, “integrated the evidence found in your expertise” item # 4 had the lowest score (mean 3.13, SD 1.1), and “sharing information with colleagues” item # 6 was observed with the highest score (mean 4.12, SD 1.5).

### Perceived barriers to EBP

**Figure 1:**
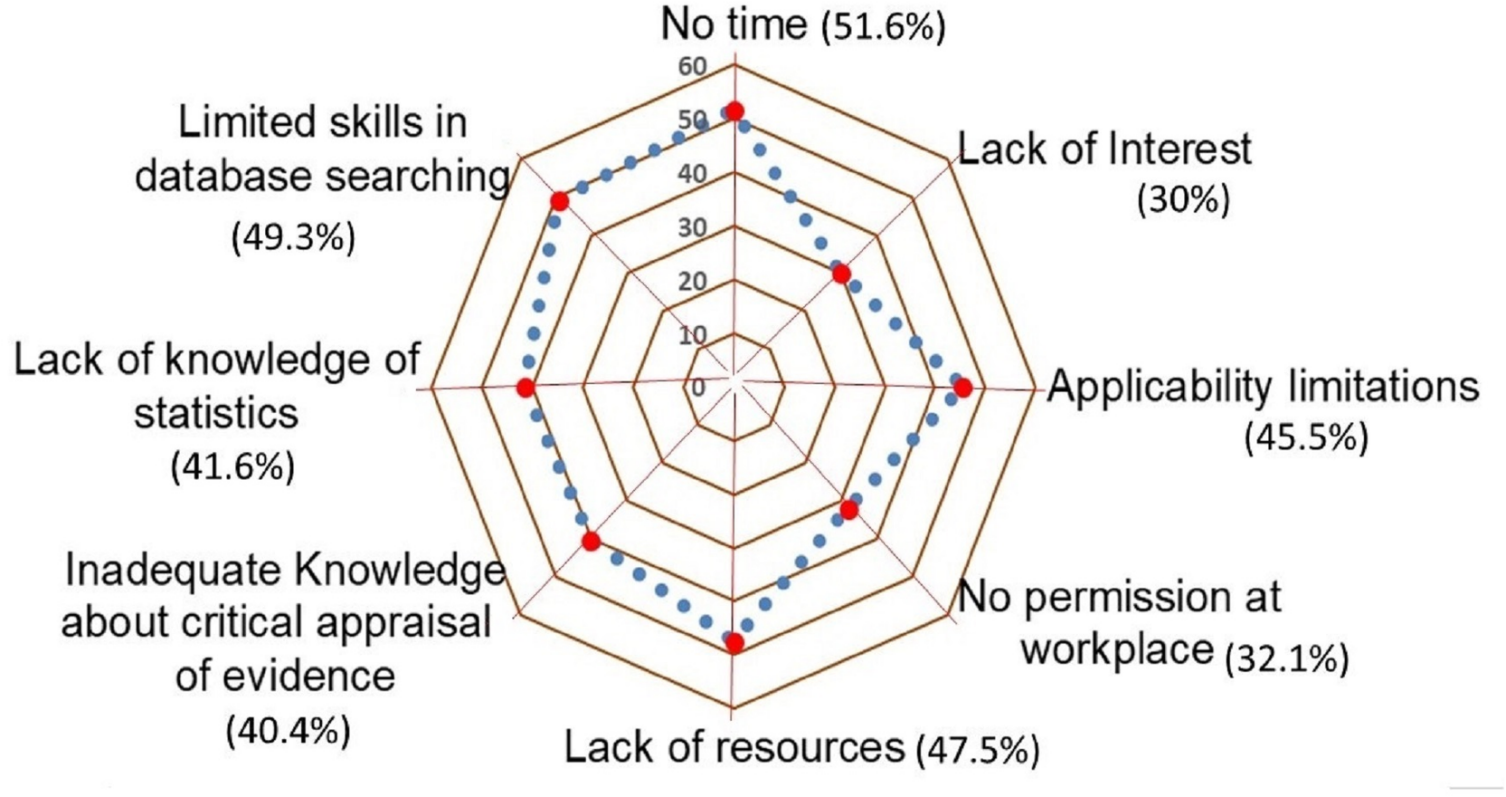
Distribution of self-reported barriers to applying EBP.

Among the provided list of perceived barriers to implementation of EBP, the participants reported lack of time (51.6%) as the greatest barrier, followed by limited skills in database searching (49.3%), lack of resources (47.5%), and limitations in clinical applicability (45.5%). And most of the participants (70%) expressed that they are interested in EBP in physiotherapy (Fig. 1).

### Regression analysis

Pearson’s correlation between attitude and EBP behavior of physiotherapists (*r* 0.251, 95% CI [0.216–0.283], *p* < 0.01) and knowledge/skills of EBP with behavior toward EBP (*r* 0.201, 95% CI [0.191–0.224], *p* < 0.01) demonstrates that a more favorable attitude and knowledge to be associated with the behavior of EBP. Furthermore, there was a negative correlation between knowledge/skills and attitude (*r* −0.101, 95% CI [−0.120 to −0.094], *p* < 0.01), suggesting that the participants with low-level knowledge reported better attitudes toward EBP (Table S3).

The standard regression coefficients suggest that the educational status and place of work were observed to be associated with the knowledge score (β 0.09 and β 0.27, respectively) and behavior score (β 0.09 and β 0.27) related to EBP among the physiotherapists. These 2 factors explained 49.6%, 50.7%, and 46.11% of the variations in the knowledge, behavior, and total EBP scores, respectively. The attitude scores are influenced by the educational status of the physiotherapist, and the model explained 41.9% variation (Table 2).

**Table 2:** Multiple linear regression for variables.

**Variables**		**Unstandardized coefficients**	**Standardized coefficients**	***t***	***p***	**95% CI for B**
Knowledge/skills R^2^49.6%		B	Beta			
Constant	3.31	0.63	54.16	<.001	3.28, 3.52
Educational status	0.04	0.08	4.50	<.001	0.02, 0.06
Workplace	0.07	0.27	14.19	<.001	0.06, 0.08
Attitude R^2^ 41.9%	Constant	3.32	0.01	51.16	0.022	3.28, 3.60
	Educational status	0.04	0.05	3.38	0.020	0.03, 0.09
Behavior/practice R^2^ 50.7%	Constant	2.85	0.60	128.27	<.001	2.78, 2.91
	Educational status	0.05	0.09	4.86	<.001	0.03, 0.07
	Work place	0.09	0.28	14.37	<.001	0.08, 0.11
EBPQ total R^2^ 46.1	Constant	3.36	0.62	115.2	<.001	3.01, 3.60
	Educational status	0.03	0.09	5.17	<.001	0.02, 0.04
	Work place	0.07	0.344	18.19	<.001	0.06, 0.07

**Notes.**

CI, Confidence interval; R^2^, R-Squared; B, regression coefficient Beta. Multiple regression analysis was performed on EBP items of knowledge (14 items) and attitude (four items) subscales to determine their influence on the EBP behavior score (Table 3). The knowledge items (X_11_ to X_24_) and attitude items (X_7_ to X_10_) were entered as independent variables, and the behavior/practice score was the dependent variable in the model. The standard regression coefficient revealed that 10 factors were finally entered into the model. Eight items of the knowledge domain and 2 items of the attitude were more influential in predicting the behavior of EBP. The 10-factor model explained 61.2% of the variation in the EBP behavior score (Adjusted *R*^2^ = 0.54).

Multiple regression analysis was performed on EBP items of knowledge (14 items) and attitude (four items) subscales to determine their influence on the EBP behavior score (Table 3). The knowledge items (X_11_ to X_24_) and attitude items (X_7_ to X_10_) were entered as independent variables, and the behavior/practice score was the dependent variable in the model. The standard regression coefficient revealed that 10 factors were finally entered in the model. Eight items from the knowledge domain and two items from the attitude domain were more influential in predicting the behavior of EBP. The 10-factor model explained 61.2% of the variation in the EBP behavior score (Adjusted *R*^2^ = 0.549).

**Table 3:** Multiple linear regression analysis of influential items of evidence-based practice.

**Variables**	**Unstandardized coefficients** **(B)**	**Standardized coefficients** **(β)**	***t***	***p***	**95% CI for B**
*Constant*	3.142	0.642	42.79	<0.001	2.99, 3.28
*Knowledge/skills domain*					
*X*_12_ *: IT skills*	.022	.046	2.21	.027	.003, .042
*X*_13_ *: Monitoring and reviewing of practice skills*	.015	.046	2.36	.018	.003, .028
*X*_14_ *: Converting your information needs into a research question*	.026	.072	3.96	<0.001	.013, .038
*X*_15_ *: Awareness of major information types and sources*	.025	.073	3.99	<0.001	.013, .035
*X*_17_ *: Knowledge of how to retrieve evidence*	.017	.036	1.75	.081	.010, .037
*X*_18_ *: Ability to analyze critically evidence against set standards*	.029	−.080	4.42	<0.001	.016, .042
*X*_19_ *: Ability to determine how useful (clinically applicable) the material is*	.028	.080	4.13	<0.001	.015, .041
*X*_22_ *: Sharing of ideas and information with colleagues*	.016	.046	2.56	.011	.004, .029
*Attitude domain*					
*X*_8_ *: I resent having my clinical practice questioned*	.034	−.095	5.08	<0.001	.021, .047
*X*_10_ *: I stick to tried and trusted methods rather than changing to anything new*	.030	−.089	4.77	<0.001	.018, .044

**Notes.**

CI Confidence interval t *t*-value R^2^ R-Squared B regression coefficient Beta

*R* = 0.612, *R*^2^ = 0.575, Adjusted *R*^2^ = 0.549, *F* = 126.4, *p* < 0.001

## Discussion

This study provided a comprehensive evaluation of EBP awareness with knowledge and implementation achievements among 2,966 physiotherapists. We observed significant differences in engagement with EBP across professional roles, regions, and educational levels. Physiotherapists employed in private practice and home care settings reported higher EBP scores than their counterparts in government hospitals. This disparity may be attributed to greater autonomy, enhanced access to treatment resources, and a stronger emphasis on patient-centred care among private and home-based practitioners. The adoption of EBP showed regional variations because Rajasthan, along with Bihar, Chhattisgarh, and Haryana, exhibited better engagement compared to Jammu & Kashmir, Himachal Pradesh, and Maharashtra. EBP implementation success depends heavily on resource availability and institutional backing, according to these identified differences. Furthermore, physiotherapists with doctoral-level education demonstrated superior EBP skills, highlighting the critical role of advanced education in developing evidence-based decision-making abilities among clinicians. Studies have demonstrated that advanced education in postgraduate programs helps healthcare providers properly understand research data alongside their capability to transform scientific evidence into clinical practice for better patient results (Hasani et al., 2020; Ashworth et al., 2014; van der Leeuw et al., 2012).

The study observed a discrepancy between positive attitudes and suboptimal implementation of EBP. While participants demonstrated favorable attitudes, their knowledge and behavioral application of EBP were moderate, aligning with global trends where enthusiasm for EBP often outstrips practical adoption (Morales-Osorio et al., 2024; Hasani et al., 2020). The regression analysis highlighted educational attainment and workplace as dominant predictors of EBP competency, collectively explaining 46.1% of the variance in total EBP scores. Private practitioners and those with advanced degrees (*e.g.*, PhDs) reported higher EBP engagement, echoing findings from similar studies in Saudi Arabia (Hasani et al., 2020). Barriers such as lack of time (51.6%), limited skills in database navigation (49.3%), and resource shortages (47.5%) were prominent, mirroring challenges identified in Latin America and in India (Morales-Osorio et al., 2024; Patel & Jhala, 2024). Further, a systematic review reporting the barriers of EBP implementation in physiotherapy involving 29 studies estimated that lack of time (53%, 95% CI [44.0–62.0]) was the most frequently reported barrier, which is consistent with the findings reported in this study (Paci et al., 2021). However, the review found that language limits and lack of access were among the top barriers. Conversely, in this study lack of skills, lack of resources, and application limitations were among the top reported barriers which is more typical of the developing countries.

Notably, the negative correlation between knowledge and attitude scores suggests that clinicians with lower EBP literacy may overestimate their readiness to implement EBP, underscoring the need for targeted educational reforms. Prospective research should examine if redesigning the undergraduate curriculum will address the EBP knowledge deficiency during initial physiotherapy education (Patel, Shah & Prakash, 2025). The adoption of EBP depended on personnel backgrounds, geographic locations, and licensing body policies, as demonstrated by the regression analysis (Gleadhill et al., 2022; Dalheim et al., 2012; Pitsillidou et al., 2021; Young & Ward, 2001). Traditional demographic factors, including clinical experience duration and age, alongside gender proved to be either minimal or sporadic in their contributions to readiness for EBP Private practice physiotherapists received more points in EBP surveys since they encounter less bureaucracy and their incentives align with staying aware of the latest evidence-based practices (Young & Ward, 2001; Al-Kubaisi, Al-Dahnaim & Salama, 2010; Bernhardsson et al., 2014). Positive attitudes toward EBP among a wide range of healthcare professionals, including nurses, physiotherapists, physicians, occupational therapists, and psychologists, have been demonstrated by previous studies (Silva et al., 2021; O’Donnell, 2004; Weng et al., 2015). Research must explore what impact private practice autonomy has on EBP adoption *versus* the barriers that government institutions raise against evidence-based practice implementation (Paci et al., 2021).

Regional discrepancies in EBP scores highlight the impact of localized factors such as infrastructure, access to training, and professional development opportunities. The moderate EBPQ scores in India reflect a broader pattern observed in low- and middle-income countries, where systemic constraints like fragmented healthcare access and under-resourced institutions hinder EBP integration (Ganesh, Khan & Khan, 2024). For instance, the emphasis on workplace settings—private clinics outperforming government hospitals—parallels findings from a Saudi Arabian study, where institutional support and autonomy facilitated EBP adoption (Dirbas & Al Haddad, 2024). The influence of advanced education aligns with global evidence that postgraduate training enhances critical appraisal skills and research utilization (Scurlock-Evans, Upton & Upton, 2014). However, India’s unique challenges, such as regional disparities (*e.g.*, low representation from Eastern states) and cultural preferences for traditional practices, necessitate context-specific strategies (Yoshikawa et al., 2020). These findings emphasize the need for region-specific capacity-building programs that integrate localized challenges such as infrastructure limitations and access to training (Sterba et al., 2020; JBI, 2024). Additionally, previous studies have demonstrated that membership in professional organizations significantly enhances EBP engagement by providing access to training and research resources (Burgers et al., 2003; Newman, Pyne & Cowling, 1998). Policymakers should consider strengthening institutional frameworks that facilitate continuous learning and professional development (Wong et al., 2021). Evaluation-based practice scores differ between regions due to specific factors that affect healthcare delivery infrastructure, together with training accessibility and professional growth opportunities (Hisham, Liew & Ng, 2018; Ambachew et al., 2023; Merza & Maani, 2023). The research demonstrates why targeted capacity-building initiatives need to develop regional programs that address the unique problems of infrastructure and training access in specific areas (Khan et al., 2018). Membership in professional organizations leads to better EBP engagement, according to research, because these organizations offer training and research assets to their members (Evans et al., 2014). Policy directors shall prioritize the establishment of institutional frameworks for professional development and continuing professional education. The identified barriers, particularly time constraints and skill gaps, resonate with barriers reported in Argentina, where physiotherapists struggled with critical appraisal and IT literacy (Bevacqua & Intelangelo, 2023; Kinney et al., 2023).

A few limitations would limit the generalizability of the findings of this study. First, the cross-sectional design does not allow us to explore the causal relationships between the identified factors and EBP engagement. Secondly, though this online survey recruited a large sample, the snowball sampling method would have led to non-representativeness, and the sample included a disproportionately high number of academic physiotherapists and variations in the geographic representation may limit the generalizability of the results to clinicians working in non-academic or clinical practice settings and under-represented regions. Third, the online format of the survey raises concerns about participant eligibility verification and the possibility of duplicate responses, the potential for social desirability bias, as well as over- or under-reporting of EBP behaviors, cannot be entirely ruled out. Finally, though this study used an established outcome tool (EBPQ), and the tool demonstrated good reliability (Cronbach’s alpha), justifying its transferability in the Indian context. Yet, it is important that country-specific factors might have influenced the scores obtained in this study, despite of the reliability reported. Hence, local contextual factors shall be considered while interpreting the findings. These limitations underscore the need for cautious interpretation and suggest that further studies using more controlled methodologies are warranted. Nevertheless, the following measures, like assuring anonymity and use of neutral language in the questions, might have reduced the desirability bias to some extent. Moreover, the larger sample-based data in this study would have enhanced the precision of the estimates reported in this study.

## Conclusion

This nationwide study underscores the multifaceted factors influencing EBP adoption among Indian physiotherapists. The findings highlight the critical role of educational background, regional disparities, and practice settings in shaping EBP implementation. Targeted strategies, including curriculum enhancements, policy reforms, and regional capacity-building initiatives, are essential to fostering a more consistent and widespread adoption of EBP in physiotherapy practice across India. To cultivate a sustainable EBP culture in Indian physiotherapy, a compassionate, multi-level approach is essential. First, educational reforms must empower future practitioners by embedding EBP modules into curricula. This will equip students not just with theoretical knowledge but with hands-on workshops to navigate databases and interpret research. Second, institutions must cultivate a culture of inquiry: hospitals could foster EBP engagement by providing free access to critical resources like the Cochrane Library and dedicated time for evidence review by clinicians. Third, respecting patients’ cultural preferences for instance, integrating evidence-based yoga into rehabilitation protocols. Finally, educators must model EBP behaviors in everyday practice, demystifying its application, while national bodies like the IAP amplify this ripple effect through structured mentorship programs.

## Supplemental Information

10.7717/peerj.20632/supp-1 Supplemental Information 1 Cherry and STROBE checklist

10.7717/peerj.20632/supp-2 Supplemental Information 2 Descriptive analysis of EBPQ items

10.7717/peerj.20632/supp-3 Supplemental Information 3 Pearson’s correlation between age, subscales of EBPQ, and overall score of EBPQ (n = 2996)

10.7717/peerj.20632/supp-4 Supplemental Information 4 Codebook of final analysis for JASP analysis for EBP

## Abbreviations

CI: Confidence Interval
EBPQ: Evidence-based practice
GDPR: General Data Protection Regulation
IAP: Indian Association of Physiotherapists
IT: Information Technology
PhD: Doctor of Philosophy
SAGER: Sex and Gender Equity in Research
SD: Standard deviation
STROBE: Strengthening the Reporting of Observational Studies in Epidemiology
UHC: Universal Health Coverage

## Additional Information and Declarations

### Data Availability

The following information was supplied regarding data availability: The data is available at figshare: Sidiq, Mohammad; Kashoo, Faizan Zaffar; Janakiraman, Balamurugan; Chahal, Aksh (2025). Final Data set for EBPQ study. figshare. Dataset. https://doi.org/10.6084/m9.figshare.28254956.v1.

### Clinical Trial Registration

The following information was supplied regarding Clinical Trial registration: CTRI/2024/09/073590.
