# Supplementary material for: Evidence-based practice among physiotherapists in India: a nationwide survey of knowledge, attitude, and implementation behavior
Source: PeerJ. 2026 Feb 4;14:e20632. doi: 10.7717/peerj.20632 (PMC12882727; doi:10.7717/peerj.20632)
Supplement: Supplemental Information 1 [file peerj-14-20632-s001.docx]

**Checklist for Reporting Results of Internet E-Surveys (CHERRIES)**

**Study title:** *Evidence-based practice among physiotherapists in India: a nationwide survey of knowledge, attitude, and implementation behavior* **, 2025**

| ***Checklist Item*** | ***Explanation*** | ***Page Number/Location*** |
| --- | --- | --- |
| Describe survey design | Describe target population, sample frame. Is the sample a convenience sample? (In “open” surveys this is most likely.) | **Page 5** *(Methods section- Target population sub-section):* Registered members of Indian Association of Physiotherapists. |
| IRB approval | Mention whether the study has been approved by an IRB. | **Page 4**, *(Methods section, Study design & registration)* Approved by IEC, Galgotias University, India (DRC/FEA/94/24), and Clinical Trial Registry India CTRI/2024/09/073590 |
| Informed consent | Describe the informed consent process. Where were the participants told the length of time of the survey, which data were stored and where and for how long, who the investigator was, and the purpose of the study? | **Page 7**, *(Methods, Ethical Considerations section).* |
| Data protection | If any personal information was collected or stored, describe what mechanisms were used to protect unauthorized access. | **Page 6***, (Methods, Data collection procedure and data storage section)* - Concealed, anonymized, and stored in an encrypted computer. Only the PI had full access. |
| Development and testing | State how the survey was developed, including whether the usability and technical functionality of the electronic questionnaire had been tested before fielding the questionnaire. | **Page 6**, *(Methods, Variables section)* - we used an EBPQ tool, developed and validated by Upton et al, 2006 |
| Open survey versus closed survey | An “open survey” is a survey open for each visitor of a site, while a closed survey is only open to a sample which the investigator knows (password-protected survey). | **Page 5**, *(Methods, Data collection procedure and data storage section)* - closed survey targeting members of the Indian Association of Physiotherapists, India. |
| Contact mode | Indicate whether or not the initial contact with the potential participants was made on the Internet. (Investigators may also send out questionnaires by mail and allow for Web-based data entry.) | Page 5, *(Methods, Data Collection Procedure, and Data Storage section)* – Invitation mail sent. |
| Advertising the survey | How/where was the survey announced or advertised? Some examples are offline media (newspapers), or online (mailing lists – If yes, which ones?) or banner ads (Where were these banner ads posted and what did they look like?). It is important to know the wording of the announcement as it will heavily influence who chooses to participate. Ideally the survey announcement should be published as an appendix. | NA |
| Web/E-mail | State the type of e-survey (eg, one posted on a Web site, or one sent out through e-mail). If it is an e-mail survey, were the responses entered manually into a database, or was there an automatic method for capturing responses? | Page 5, *(Methods, Data Collection Procedure, and Data Storage section), responses manual entered* |
| Context | Describe the Web site (for mailing list/newsgroup) in which the survey was posted. What is the Web site about, who is visiting it, what are visitors normally looking for? Discuss to what degree the content of the Web site could pre-select the sample or influence the results. For example, a survey about vaccination on a anti-immunization Web site will have different results from a Web survey conducted on a government Web site | **Page 6**, *(Methods, Variables section)* |
| Mandatory/voluntary | Was it a mandatory survey to be filled in by every visitor who wanted to enter the Web site, or was it a voluntary survey? | **Page 7**, *(Methods, Ethical Considerations section). A voluntary survey was used* |
| Incentives | Were any incentives offered (eg, monetary, prizes, or non-monetary incentives such as an offer to provide the survey results)? | **Page 7**, *(Methods, Ethical Considerations section).* No incentives or compensation were provided |
| Time/Date | In what timeframe were the data collected? | **Page 5**, *(Methods, Data Collection Procedure, and Data Storage section),* Survey period (15 weeks period from Sept 2024 to December 2024) |
| Randomization of items or questionnaires | To prevent biases items can be randomized or alternated. | Not used |
| Adaptive questioning | Use adaptive questioning (certain items, or only conditionally displayed based on responses to other items) to reduce number and complexity of the questions. | NA |
| Number of Items | What was the number of questionnaire items per page? The number of items is an important factor for the completion rate. | **Page 6**, *(Methods, Variables section),*  ‘6 - 8’ questions per page. |
| Number of screens (pages) | Over how many pages was the questionnaire distributed? The number of items is an important factor for the completion rate. | **Page 6**, *(Methods, Variables section),*  7 pages |
| Completeness check | It is technically possible to do consistency or completeness checks before the questionnaire is submitted. Was this done, and if “yes”, how (usually JAVAScript)? An alternative is to check for completeness after the questionnaire has been submitted (and highlight mandatory items). If this has been done, it should be reported. All items should provide a non-response option such as “not applicable” or “rather not say”, and selection of one response option should be enforced. | **Page 6**, *(Methods, Variables section),*  we used highlights for mandatory items. |
| Review step | State whether respondents were able to review and change their answers (eg, through a Back button or a Review step which displays a summary of the responses and asks the respondents if they are correct). | **Page 6**, *(Methods, Variables section) -* yes, the respondents were allowed to review and change their responses. |
| Unique site visitor | If you provide view rates or participation rates, you need to define how you determined a unique visitor. There are different techniques available, based on IP addresses or cookies or both. | No, we did not |
| View rate (Ratio of unique survey visitors/unique site visitors) | Requires counting unique visitors to the first page of the survey, divided by the number of unique site visitors (not page views!). It is not unusual to have view rates of less than 0.1 % if the survey is voluntary. | NA |
| Participation rate (Ratio of unique visitors who agreed to participate/unique first survey page visitors) | Count the unique number of people who filled in the first survey page (or agreed to participate, for example by checking a checkbox), divided by visitors who visit the first page of the survey (or the informed consents page, if present). This can also be called “recruitment” rate. | Yes, reported |
| Completion rate (Ratio of users who finished the survey/users who agreed to participate) | The number of people submitting the last questionnaire page, divided by the number of people who agreed to participate (or submitted the first survey page). This is only relevant if there is a separate “informed consent” page or if the survey goes over several pages. This is a measure for attrition. Note that “completion” can involve leaving questionnaire items blank. This is not a measure for how completely questionnaires were filled in. (If you need a measure for this, use the word “completeness rate”.) | NA |
| Cookies used | Indicate whether cookies were used to assign a unique user identifier to each client computer. If so, mention the page on which the cookie was set and read, and how long the cookie was valid. Were duplicate entries avoided by preventing users access to the survey twice; or were duplicate database entries having the same user ID eliminated before analysis? In the latter case, which entries were kept for analysis (eg, the first entry or the most recent)? | NA |
| IP check | Indicate whether the IP address of the client computer was used to identify potential duplicate entries from the same user. If so, mention the period of time for which no two entries from the same IP address were allowed (eg, 24 hours). Were duplicate entries avoided by preventing users with the same IP address access to the survey twice; or were duplicate database entries having the same IP address within a given period of time eliminated before analysis? If the latter, which entries were kept for analysis (eg, the first entry or the most recent)? | The survey response is limited to the mail IDs, each mail ID is allowed one response. |
| Log file analysis | Indicate whether other techniques to analyze the log file for identification of multiple entries were used. If so, please describe. | NA |
| Registration | In “closed” (non-open) surveys, users need to login first and it is easier to prevent duplicate entries from the same user. Describe how this was done. For example, was the survey never displayed a second time once the user had filled it in, or was the username stored together with the survey results and later eliminated? If the latter, which entries were kept for analysis (eg, the first entry or the most recent)? | NA |
| Handling of incomplete questionnaires | Were only completed questionnaires analyzed? Were questionnaires which terminated early (where, for example, users did not go through all questionnaire pages) also analyzed? | We used completed responses only. |
| Questionnaires submitted with an atypical timestamp | Some investigators may measure the time people needed to fill in a questionnaire and exclude questionnaires that were submitted too soon. Specify the timeframe that was used as a cut-off point, and describe how this point was determined. | **Page 5**, *(Methods, Data Collection Procedure, and Data Storage section), Closing date, 31* December 2024 |
| Statistical correction | Indicate whether any methods such as weighting of items or propensity scores have been used to adjust for the non-representative sample; if so, please describe the methods. | **NA** |

This checklist has been modified from Eysenbach G. Improving the quality of Web surveys: the Checklist for Reporting Results of Internet E-Surveys (CHERRIES). J Med Internet Res. 2004 Sep 29;6(3):e34 [erratum in J Med Internet Res. 2012; 14(1): e8.].

Article available at [https://www.jmir.org/2004/3/e34](https://www.jmir.org/2004/3/e34/)/; erratum available <https://www.jmir.org/2012/1/e8/>. Copyright ©Gunther Eysenbach. Originally published in the [Journal of Medical Internet](http://www.jmir.org) Research, 29.9.2004 and 04.01.2012.

This is an open-access article distributed under the terms of the Creative Commons Attribution License (<https://creativecommons.org/licenses/by/2.0/>), which permits unrestricted use, distribution, and reproduction in any medium, provided the original work, first published in the Journal of Medical Internet Research, is properly cited.

**STROBE checklist for the study:** *Evidence-based practice among physiotherapists in India: a nationwide survey of knowledge, attitude, and implementation behavior***, 2025**

|  | Item No. | Recommendation | Page  No. | Relevant text from manuscript |
| --- | --- | --- | --- | --- |
| **Title and abstract** | 1 | (*a*) Indicate the study’s design with a commonly used term in the title or the abstract | Page 1 | A web-based cross-sectional survey |
|  |  | (*b*) Provide in the abstract an informative and balanced summary of what was done and what was found | Page 1 | *Knowledge or skill, attitude, and behavior towards implementation of evidence-based practice (EBP) in physiotherapy care were studied.*  *The study observed a positive attitude and insufficient knowledge.* |
| Introduction | | | |  |
| Background/rationale | 2 | Explain the scientific background and rationale for the investigation being reported | Page 3 | Literature report favourable attitude, limited knowledge, and barriers to implementation of EBP. But, a context specific investigation is warranted to inform policy. |
| Objectives | 3 | State specific objectives, including any pre-specified hypotheses | Page 3 - 4 | *We hypothesize that there is a positive relationship between the knowledge and attitude towards evidence-based practice and implementation behaviour among physiotherapists in India.* |
| Methods | | | |  |
| Study design | 4 | Present key elements of study design early in the paper | Page 4 | Web-based cross-sectional survey |
| Setting | 5 | Describe the setting, locations, and relevant dates, including periods of recruitment, exposure, follow-up, and data collection | Page 5 | Reported under the Methods section |
| Participants | 6 | *Cross-sectional study*—Give the eligibility criteria, and the sources and methods of selection of participants | Page 5 | Eligibility criteria para, under Study setting and participants |
| Variables | 7 | Clearly define all outcomes, exposures, predictors, potential confounders, and effect modifiers. Give diagnostic criteria, if applicable | Page 6 | Variables: Socio-demographic, work related characteristics, and 24 item EBP questionnaire. |
| Data sources/ measurement | 8* | For each variable of interest, give sources of data and details of methods of assessment (measurement). Describe comparability of assessment methods if there is more than one group | Page 6-7 | *Self-reported outcome measures* |
| Bias | 9 | Describe any efforts to address potential sources of bias | Page 14 | Desirability bias: measures taken to ensure anonymity, confidentiality assurance, and use indirect questions. |
| Study size | 10 | Explain how the study size was arrived at | Page 7-8 | Section : Sample size and post-hoc power analysis |
| Quantitative variables | 11 | Explain how quantitative variables were handled in the analyses. If applicable, describe which groupings were chosen and why | Page 8 | Reported in Data analysis section |
| Statistical methods | 12 | (*a*) Describe all statistical methods, including those used to control for confounding | Page 8 | Reported in Data analysis section |
|  |  | (*b*) Describe any methods used to examine subgroups and interactions | Page 8 |  |
|  |  | (*c*) Explain how missing data were addressed | Page 8 |  |
|  |  | (*d*) If applicable, describe analytical methods taking account of sampling strategy | NA |  |
|  |  | (*e*) Describe any sensitivity analyses | NA |  |
| Results |  |  |  |  |
| Participants | 13* | (a) Report numbers of individuals at each stage of study—eg numbers potentially eligible, examined for eligibility, confirmed eligible, included in the study, completing follow-up, and analysed | Page 9 |  |
|  |  | (b) Give reasons for non-participation at each stage | NA |  |
|  |  | (c) Consider use of a flow diagram | NA |  |
| Descriptive data | 14* | (a) Give characteristics of study participants (eg demographic, clinical, social) and information on exposures and potential confounders | Page 10 |  |
|  |  | (b) Indicate number of participants with missing data for each variable of interest | NA |  |
| Outcome data | 15* | Report numbers of outcome events or summary measures | Page 10 |  |
| Main results | 16 | (*a*) Give unadjusted estimates and, if applicable, confounder-adjusted estimates and their precision (eg, 95% confidence interval). Make clear which confounders were adjusted for and why they were included | Page 10 -11 |  |
|  |  | (*b*) Report category boundaries when continuous variables were categorized | Page 11 |  |
|  |  | (*c*) If relevant, consider translating estimates of relative risk into absolute risk for a meaningful time period | NA |  |
| Other analyses | 17 | Report other analyses done—eg analyses of subgroups and interactions, and sensitivity analyses | NA |  |
| **Discussion** |  |  |  |  |
| Key results | 18 | Summarise key results with reference to study objectives | Page 11 |  |
| Limitations | 19 | Discuss limitations of the study, taking into account sources of potential bias or imprecision. Discuss both direction and magnitude of any potential bias | Page 13 |  |
| Interpretation | 20 | Give a cautious overall interpretation of results considering objectives, limitations, multiplicity of analyses, results from similar studies, and other relevant evidence | Page 13 |  |
| Generalisability | 21 | Discuss the generalisability (external validity) of the study results | Page 13 |  |
| Other Informations |  |  |  |  |
| Funding | 22 | Give the source of funding and the role of the funders for the present study and, if applicable, for the original study on which the present article is based | Under funding section |  |
|  |  |  |  |  |

Continued on next page
