# Supplementary material for: Evidence-based practice among physiotherapists in India: a nationwide survey of knowledge, attitude, and implementation behavior
Source: PeerJ. 2026 Feb 4;14:e20632. doi: 10.7717/peerj.20632 (PMC12882727; doi:10.7717/peerj.20632)
Supplement: Supplemental Information 2 [file peerj-14-20632-s002.docx]

**Table 1**

**Sex-wise distribution of socio-demographic variables and EBPQ scores**

| **Characteristics** | **Descriptive statistics** | | |
| --- | --- | --- | --- |
|  | **Overall** | **Female**  **n 1872** | **Male**  **n 1124** |
| ^⸸^Age in years, mean ± SD | 35.83 ± 6.22 | 35.88±6.24 | 35.74±6.19 |
| Age group, n (%) |  |  |  |
| < 30 years | 648 (21.6) | 405 (21.6) | 243 (21.6) |
| 30 – 40 years | 1792 (59.8) | 1106 (59.1) | 686 (61) |
| >40 years | 556 (18.6) | 361 (19.3) | 195 (17.3) |
| Geographical zones, India, n (%) |  |  |  |
| Central zone | 713 (23.8) | 437 (23.3) | 276 (24.6) |
| Eastern zone | 09 (0.3) | 5 (0.3) | 5 (0.4) |
| Northern zone | 1113 (37.1) | 713 (38.1) | 399 (35.5) |
| Southern zone | 768 (25.6) | 471 (25.2) | 296 (26.4) |
| Western zone | 393 (13.2) | 246 (13.1) | 147 (13.1) |
| Educational status |  |  |  |
| Diploma | 7 (0.2) | 5 (0.3) | 2 (0.2) |
| Bachelor’s degree | 1225 (40.9) | 798 (42.6) | 427 (38) |
| Master’s degree | 1470 (49.1) | 899 (48) | 571 (50.8) |
| DPT | 22 (0.7) | 11 (0.6) | 11 (1) |
| PhD scholar | 11 (0.4) | 8 (0.4) | 3 (0.3) |
| PhD degree | 261 (8.7) | 151 (8.1) | 110 (9.8) |
| Work place, n (%) |  |  |  |
| Community care | 669 (22.3) | 402 (21.5) | 267 (23.8) |
| Private clinic | 341 (11.4) | 226 (12.1) | 115 (10.2) |
| Nursing home/poly clinic | 335 (11.2) | 220 (11.8) | 115 (10.2) |
| Hospital | 538 (18) | 332 (17.7) | 206 (18.3) |
| Teaching Hospital/university | 380 (12.7) | 241 (12.9) | 139 (12.4) |
| Private college | 733 (24.5) | 451 (24.1) | 282 (25.1) |
| ^⸸^Work experience, mean ± SD | 13.25 ± 6.38 | 13.29 ±6.4 | 13.10 ±6.3 |
| ^⸸^Knowledge dimension score | 50.24±7.4 | 50.26±7.3 | 50.18±7.4 |
| ^⸸^Attitude dimension score | 17.15±3.17 | 17.16±3.2 | 17.14±3.2 |
| ^⸸^Practice dimension score | 19.18±3.72 | 19.12±3.7 | 19.27±3.7 |
| Overall EBPQ score (24-168) | 86.57±9.13 | 86.55±9.21 | 86.59±9.1 |
| ^⸸^Knowledge subscale (1 - 7) | 3.59 ±0.53 | 3.59 ±0.53 | 3.58±0.53 |
| ^⸸^Attitude subscale (1 - 7) | 4.29 ±0.79 | 4.28 ±0.80 | 4.29±0.78 |
| ^⸸^Practice subscale (1 - 7) | 3.20 ±0.62 | 3.19 ±0.62 | 3.19±0.62 |
| ^⸸^EBPQ overall (1 - 7) | 3.61 ±0.38 | 3.61 ±0.38 | 3.61±0.38 |

^⸸^Expressed as mean and standard deviation and analyzed using student t test and Chisquare test for categorical variables

**Table 2 Descriptive analysis of EBPQ items**

| **Dimension** | **Item** | **Statement** | **Mean ±** |
| --- | --- | --- | --- |
| **Knowledge/skills** | 1 | Research skills | 3.64±1.9 |
|  | 2 | IT skills | 3.28±1.3 |
|  | 3 | Monitoring and reviewing of practice skills | 4.77±1.9 |
|  | 4 | Converting your information needs into a research question | 3.89±1.7 |
|  | 5 | Awareness of major information types and sources | 4.51±1.8 |
|  | 6 | Ability to identify gaps in your professional practice | 3.49±1.9 |
|  | 7 | Knowledge of how to retrieve evidence | 3.28±1.3 |
|  | 8 | Ability to analyze critically evidence against set standards | 4.24±1.7 |
|  | 9 | Ability to determine how valid (close to the truth) the material is | 3.66±1.4 |
|  | 10 | Ability to determine how useful (clinically applicable) the material is | 4.62±1.7 |
|  | 11 | Ability to apply information to individual cases | 3.43±1.8 |
|  | 12 | Sharing of ideas and information with colleagues | 3.85±1.8 |
|  | 13 | Dissemination of new ideas about care to colleagues | 3.70±1.6 |
|  | 14 | Ability to review your own practice | 4.19±1.9 |
| **Attitude** | 1 | New evidence is important | 3.85±1.4 |
|  | 2 | I welcome questions on my practice | 4.59±1.7 |
|  | 3 | EBP is fundamental to professional practice | 4.2±1.0 |
|  | 4 | My practice has changed because of evidence | 4.51±1.8 |
| **Practice** | 1 | Formulated a clearly answerable question to fill the gap | 3.40±1.1 |
|  | 2 | Tracked down the relevant evidence | 4.01±1.7 |
|  | 3 | Critically appraised literature, against set criteria | 3.66±1.2 |
|  | 4 | Integrated the evidence you found with your expertise | 3.13±1.1 |
|  | 5 | Evaluated the outcome of your practice | 3.43±1.0 |
|  | 6 | Shared this information with colleagues | 4.12±1.5 |

**± - Standard deviation**

**Table 3**

**Pearson’s correlation between age, subscales of EBPQ, and overall score of EBPQ (n = 2996)**

| **Variables** | **Age**  **r (95% CI)** | **Practice (6 items) EBPQ**  **r (95% CI)** | **Attitude (4 items) EBPQ**  **r (95% CI)** | **Knowledge (14 items) EBPQ**  **r (95% CI)** |
| --- | --- | --- | --- | --- |
| Age | -- |  |  |  |
| Practice (6 items) EBPQ | 0.024 (-0.08, 0.051) | -- |  |  |
| Attitude (4 items) EBPQ | 0.009 (-0.02, 0.022) | **.251^**^ (0.216, 0.283)** | -- |  |
| Knowledge (14 items) EBPQ | -0.011 (-0.038, 0.008) | **.201^**^ (0.191, 0.224)** | **-.101^**^ (-0.120, -0.094)** | -- |
| EBPQ Total | 0.013 (0.004, 0.046) | **.510^**^ (0.485, 0.538)** | **.265^**^ (0.199, 0.290)** | **.862^**^ (0.643, 0.891)** |

**Correlation is significant at the 0.01 level (2-tailed). CI- Confidence Interval
