# Supplementary material for: Evidence-based practice among physiotherapists in India: a nationwide survey of knowledge, attitude, and implementation behavior
Source: PeerJ. 2026 Feb 4;14:e20632. doi: 10.7717/peerj.20632 (PMC12882727; doi:10.7717/peerj.20632)
Supplement: Supplemental Information 3 [file peerj-14-20632-s003.docx]

**Table 3**

**Pearson’s correlation between age, subscales of EBPQ, and overall score of EBPQ (n = 2996)**

| **Variables** | Age | Practice (6 items) EBPQ | Attitude (4 items) EBPQ | Knowledge (14 items) EBPQ |
| --- | --- | --- | --- | --- |
| Age | -- |  |  |  |
| Practice (6 items) EBPQ | 0.024 | -- |  |  |
| Attitude (4 items) EBPQ | 0.009 | **.251^**^** | -- |  |
| Knowledge(14 items) EBPQ | -0.011 | **.201^**^** | **-.101^**^** | -- |
| EBPQ Total | 0.013 | **.510^**^** | **.265^**^** | **.862^**^** |

**Correlation is significant at the 0.01 level (2-tailed).
